# Chronic high dose of captopril induces depressive-like behaviors in mice: possible mechanism of regulatory T cell in depression

**DOI:** 10.18632/oncotarget.19879

**Published:** 2017-08-03

**Authors:** Hyun-Sun Park, Arum Han, Hye-Lim Yeo, Min-Jung Park, Min-Jung You, Hyun Jin Choi, Chang-Won Hong, Sang-Hyuk Lee, Seung Hyun Kim, Borah Kim, Min-Soo Kwon

**Affiliations:** ^1^ Department of Pharmacology, School of Medicine, CHA University, Seongnam-si, Gyeonggi-do, Republic of Korea; ^2^ Department of Psychiatry, CHA Bundang Medical Center, CHA University, Seongnam-si, Gyeonggi-do, Republic of Korea; ^3^ College of Pharmacy, CHA University, Seongnam-si, Gyeonggi-do, Republic of Korea; ^4^ Cell Therapy Center and Department of Neurology, College of Medicine, Hanyang University, Haengdang-dong, Seoul, Republic of Korea; ^5^ Department of Physiology, School of Medicine, Kyungpook National University, Daegu, Gyeongbuk, Republic of Korea

**Keywords:** depression, regulatory T cell, cytokines, angiotensin II, captopril

## Abstract

Major depression has various types of symptoms and disease courses with inconsistent response to monoamine-related antidepressants. Thus, monoamine theory may not be the only pathophysiologic pathway relevant to depression. Recently, it has been suggested that regulatory T cell (Treg) is associated with depression. Based on our previous study that showed decreased regulatory T cell (Treg) population following chronic high-dose captopril (CHC, 40 mg/kg/day * 21 days) administration, we examined whether CHC alone can induce depressive-like behaviors in mice even without stressful stimuli. In this study, we found that CHC induced depressive-like behaviors in tail suspension test (TST) and forced swimming test (FST) without systemic illness, while it did not induce anhedonic behavior, anxiety-like behaviors, or sociality-related behavior. The depressive-like behaviors were rescued by either CHC washout or antidepressant. CHC caused reduction in foxp3 and gata3 mRNA expression in the lymph nodes with elevation in plasma IL-1β and IL-6. Interestingly, CHC increased serum angiotensin II level. In the hippocampus, CHC increased TNF-α and IL-6 mRNA expression with microglia activation while reduced glucocorticoid receptor expression. However, CHC did not affect to hippocampal kynurenine pathway, serotonin level, hypothalamic corticotropin-releasing hormone mRNA level, or serum corticosterone level. Consequently, we propose that CHC may induce a specific form of depressive-like behaviors via Treg reduction and microglial activation.

## INTRODUCTION

Major depression is one of the most disabling and chronic psychiatric diseases in the modern society. Global Burden of Disease studies from 1990, 2000 and 2010 has identified depression as one of the leading causes with disability and a risk factor that contributes to suicide and ischemic heart disease [1, 2].

Nevertheless, current treatment for depression is still based on “monoamine hypothesis” which bears several limitations. The monoamine hypothesis is based on the findings that antidepressants, such as tricyclic antidepressants (TCAs) and norepinephrine/serotonin reuptake inhibitors (N/SRI), can enhance monoamine levels in the CNS [3]. However, monoamine hypothesis alone appears to be insufficient to completely expain the depression pathophysiology due to some limitations such as non-responsiveness, recurrence and side effects [4, 5]. Thus, there is an urgent necessity to identify new pathophysiologic target to develop new medications for depression.

Recently, “cytokine theory” was emerged and attempted to explain pathomechanism of depressive disorder with immunologic factors [6, 7]. There are several evidences that elevated serum and CNS pro-inflammatory cytokines, such as IL-1β, IL-6, IFN-γ, and TNF-α or increased inflammation response are associated with depressive symptom or severity of disease [8–11]. These factors not only serve a role as mediators of peripheral inflammation but also communicate with the CNS as an inflammation inducer in the CNS. Kynurenine (KYN) pathway has been suggested as a factor linking “cytokine theory” and “monoamine theory” in depression. The enzymes involved in KYN pathway, such as indoleamine 2,3-dioxygenase (IDO), kynurenine aminotransferase (KAT) and kynurenine monooxygenase (KMO, sometimes referred as kynurenine hydroxylase) are strongly associated with cytokines [7, 12]. Activation of KYN pathway by pro-inflammatory cytokines induces the degradation of tryptophan, finally leading to serotonin depletion [7]. In addition, quinolinic acid, a constituent of KYN pathway, was also found to act as NMDA receptor agonist and exhibited depressive features, such as decreased reaction time and difficulties in learning [13]. Thus, change in circulating and the CNS cytokines may be additional candidates involved in depression pathophysiology.

Microglia, innate immune cells and major cytokines producer in the CNS, are found to communicate with the peripheral immune system and may be associated with depressive disorder [14, 15]. Studies using positron emission tomography (PET) imaging showed microglial activation in depressed patients, who committed suicide [16, 17]. Moreover, it is reported that lipopolysaccharide (LPS), which can activate microglia, can also induce depressive-like symptoms [18]. The functional phenotype of microglia is also involved in depression and stress vulnerability [19, 20]. These results suggest the possibility that factors associated with regulation of microglial function, such as cytokines [21], angiotensin II (ANG II) [22] and endothelin [23], can be hints to solve the complicated pathophysiology of major depressive disorder.

Among peripheral immune cells, regulatory T cells (Treg, CD4+ CD25+ Foxp3+) have been known to modulate the immune system, including functional phenotypes of microglia [24, 25], and they exhibit neuroprotective effects by inhibiting inappropriate or excessive immune responses [26, 27]. In addition, we reported that T-cell depleted nude mice and chronically restraint stressed mice with Treg and T Helper 2 cells (Th2) reduction exhibited stress vulnerability with microglia dysfunction, which may be associated with depression recurrence [19]. Treg depletion with anti-CD25 antibody in mice also could contribute to depressive symptoms [26]. Furthermore, anti-inflammatory cytokines, which can be released by Treg, block the development of endotoxin-induced behavioral alterations [13]. Thus, it is speculated that peripheral Treg dysfunction may possibly be involved in depression by affecting microglia functional phenotypes [28].

Captopril is angiotensin converting enzyme inhibitor that suppresses conversion of angiotensin I to angiotensin II. Also, captopril is used for hypertension treatment by suppressing renin-angiotensin-aldosterone system (RAAS) that is known to regulate blood pressure. In our previous study, we found that chronic high-dose captopril (CHC) decreased intratumoral CD4+ CD25+ Foxp3+ Treg [29], and this finding may be independent of previously known function of captopril. Based on several papers that reported a possible association between decreased Treg population and depression patients [30–32], we hypothesized that CHC may induce a specific form of depressive-like behaviors via Treg reduction and microglial activation.

## RESULTS

### Chronic high dose captopril (CHC) induced depressive-like behaviors in TST and FST

**Figure 1:**
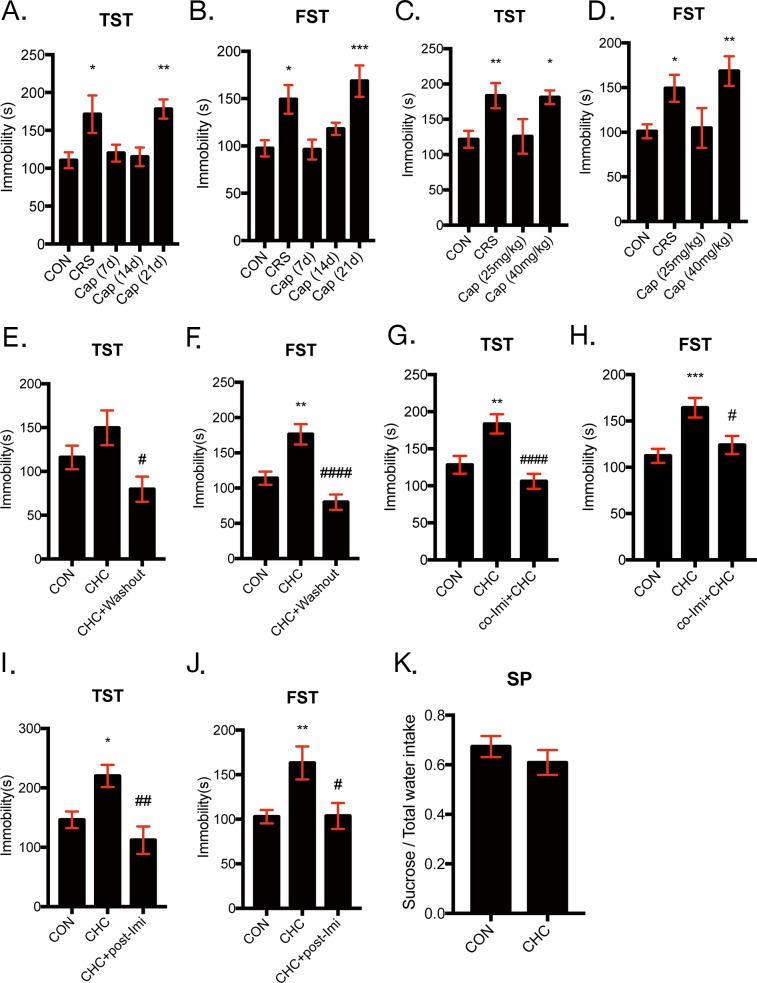
Chronic high-dose captopril (CHC) induced depressive-like behavior in mice The time-course effect and dose-dependent effect of captopril were examined by observing depression-like behavior in mice by TST and FST. Mice were administrated with captopril (40 mg/kg) for 7, 14, 21 days and their behavior was assessed with TST **(A)** and FST **(B)** and n = 10 - 20 in each group. Mice were also administrated with 25 or 40 mg/kg of captopril for more than 21 days and their behavior was assessed with **(C)** TST and **(D)** FST and n = 10 - 20 in each group. Chronic high-dose captopril (CHC) is defined as 40 mg/kg of captopril in drinking water for 21 days or more and all behavior tests were performed within a week after CHC administration. Captopril administration was continued during behavioral test. The effect of captopril washout for 7 days after the end of CHC was assessed by TST **(E)** and FST **(F)** and n = 10 - 15 in each group. The effect of imipramine co-treatment (Co-Imi) during CHC was assessed by TST **(G)** and FST **(H)** and n = 22 – 27 in each group. The effect of imipramine post-treatment (Post-Imi) was assessed by TST **(I)** and FST **(J)** and n = 7 - 14 in each group. Sucrose preference test (SP) for 1 % sucrose solution over regular drinking water was examined for 2 days after 2 days of inhabitation to two bottle conditions **(K)**. The data shown are mean ± SEM. * p < 0.05, ** p < 0.01, *** p < 0.001 compared with the controls. # p < 0. 05, ## p < 0.01, #### p < 0.0001 compared with the CHC- treated groups.

Based on previous studies about association between the reduction in Treg population with depressive-like behaviors and studies that found chronic high dose captopril (CHC) can reduce Treg population, series of experiments to identify the effect of captopril on mice behavior were performed. Captopril at concentration of 40 mg/kg was administered to mice by dissolving in drinking water for 7, 14, and 21 days and we performed TST and FST to confirm time-course effect of high dose of captopril on depressive-like behavior of mice. Both FST and TST are the most frequently used tests to evaluate the depression-like behaviors in rodents [33]. In both FST and TST, mice encounter an inescapable stress and the elevation of immobility time means helpless immobile behaviors, which reflects depressive-like mood of mice. Captopril-treated mice began to show depressive-like behaviors after 21 days of high dose captopril administration in TST and FST and the immobility time observed in the CHC mice was similar to that of CRS mice (Figure 1A, 1B). We also examined dose-dependent effect of captopril on depressive-like behaviors. Figure 1C and 1D showed that 40 mg/kg of captopril increased immobility time in both of TST and FST, while 25 mg/kg of captopril did not alter the immobility time. Hence, chronic high-dose of captopril treatment (CHC) was defined as administering 40 mg/kg of captopril for more than 21 days and CHC increased depressive-like behaviors of mice in TST and FST. Captopril washout for 7 days after CHC discontinuation restored depression-like behaviors in TST and FST (Figure 1E, 1F). To evaluate antidepressant effect of imipramine on CHC-induced depressive-like behavior, tricyclic antidepressant imipramine was treated during CHC administration or after induction of depressive-like behaviors by CHC. The increased immobility time caused by CHC was attenuated by co-treatment of imipramine with CHC in both of TST and FST (Figure 1G, 1H). To evaluate antidepressant effect of imipramine after induction of depression-like behavior by CHC, imipramine was treated for 14 days with additional captopril after 21 days of captopril treatment and this procedure is referred to as ‘post-treatment of imipramine’. During the post-treatment imipramine experiment, captopril in drinking water was administered continuously because captopril washout normalizes immobility time in TST and FST. Post-treatment of imipramine for 14 days to the CHC mice restored increased immobility time in both TST and FST (Figure 1I, 1J). In addition, the anhedonic behavior of mice was assessed by sucrose preference test. Sucrose preference test (SP) is well-established behavior test to examine depressive-like symptom, especially anhedonia, with TST and FST [34]. Interestingly, CHC-mice did not exhibit anhedonic behavior (Figure 1K).

### Chronic high dose captopril (CHC) did not affect to anxiety and sociality in mice

The behaviors associated with anxiety and sociality were assessed by EPM, LD, and social interaction test. In contrast to depressive-like behaviors, CHC did not affect anxiety and sociality in mice. There were no significant differences between the controls and CHC groups in EPM, LD, and SI (Supplementary Figure 2A-2C).

In order to investigate the possible toxicity induced from CHC, we examined general condition of mice by measuring body weight, serum GOT and evaluating motor activity by rotarod test. The body weight was decreased in CHC mice in agreement with previous study [35] and was increased slightly by captopril washout (Supplementary Figure 2D). There were no differences between the controls and CHC mice in serum GOT level and motor activity (Supplementary Figure 2E, 2F).

### Chronic high dose captopril (CHC) decreased regulatory T cell (Treg) populations in the spleen and affected the peripheral immune system

**Figure 2:**
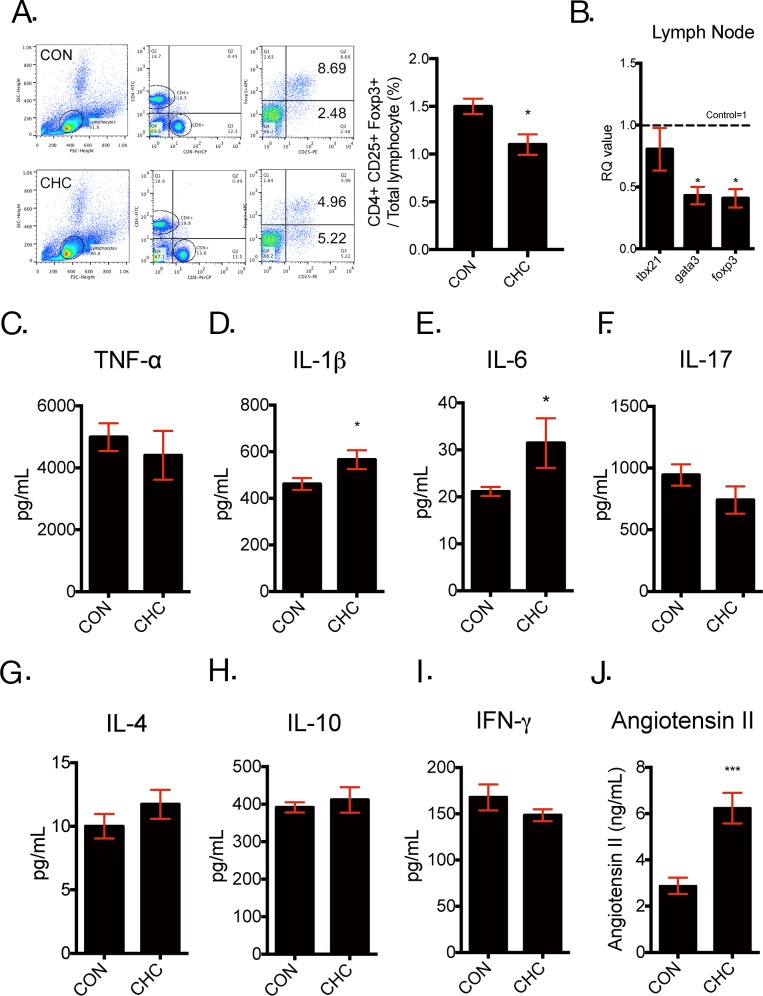
CHC-induced depressive-like behavior is associated with altered peripheral immune system Mice were administrated with CHC (40 mg/ kg/ 21 days or more). Following the termination of CHC treatment, spleens of mice were analyzed with flow cytometry and expressed with percentage of CD4+ CD25+Foxp3+ cells to total lymphocytes **(A)** and n = 3 in each group. Mesenteric lymph nodes of mice were also dissected for quantitative reverse transcriptase polymerase chain reactions(qPCR) to assess transcription factors of T Helper 1 cells (tbx21), T Helper 2 cells (gata3) and regulatory T cells (foxp3) **(B)**. n= 8 – 15 in each group. Serums of CHC mice and the controls were obtained to measure pro- and anti-inflammatory cytokines, such as TNF-α **(C)**, IL-1β **(D)**, IL-6 **(E)**, IL-17 **(F)**, IL-4 **(G)**, IL-10 **(H)**, IFN-γ**(I)**, using Bio-Rad Bio-Plex® assay and circulating levels of angiotensin II **(J)** by Enzyme Immunoassay (EIA). n = 8 – 10 in each group. The data shown are mean ± SEM.* p < 0.05, *** p < 0.001 compared with the controls.

To confirm the effect of CHC on Treg population, Treg population in mice was measured by flow cytometry. In accordance with our previous study [29], CHC decreased Treg population in the spleen (Figure 2A). In addition, peripheral inflammation was found to have possibility to induce depressive symptoms [6, 26, 36, 37]. Thus, we analyzed mRNA markers related to various subtypes of T cells in the mesenteric lymph nodes and serum cytokine profile associated with inflammation. The mRNA expressions of gata3 and foxp3, which are markers of Th2 and Treg respectively, were decreased in the mesenteric lymph nodes of CHC mice and there were no significant alterations in tbx21 (T Helper 1 cells) mRNA expressions (Figure 2B). In addition, CHC increased circulating IL-1β and IL-6 levels (Figure 2D, 2E), while there were no alterations in other inflammatory cytokines such as TNF-α, IL-17, IL-4, IL-10 and IFN-γ (Figure 2C, 2F-2I). Also, serum angiotensin II (ANG II) level was measured to investigate the effect of CHC on ANG II. However, serum ANG II levels in CHC mice were increased in CHC mice in spite of inhibition effect of captopril on angiotensin converting enzyme (ACE) (Figure 2J).

### CHC mice exhibited increased microglial activation in the hippocampus but not in the hypothalamus

**Figure 3:**
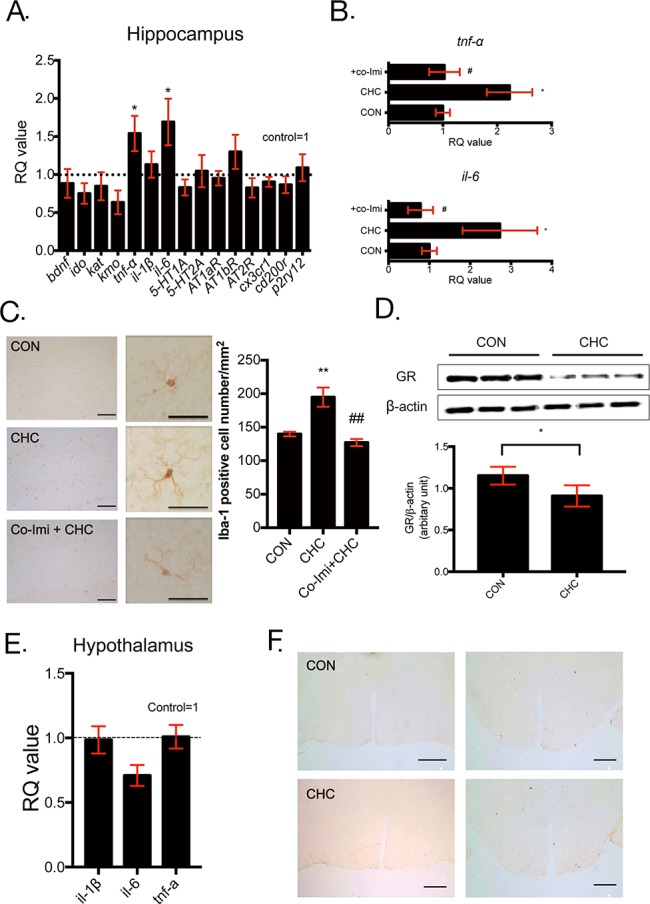
CHC increased inflammatory reaction in the hippocampus but not in the hypothalamus Several markers involved in depression (bdnf, ido, kat, kmo), pro-inflammatory cytokines (tnf-α, il-1β, and il-6), serotonin receptors (5-HT1A and 5-HT2A), angiotensin receptors (AT1bR, AT1bR, and AT2R), and microglial phenotype (cx3cr1, cd200r, and p2ry12) were examined in the hippocampus **(A)** and n = 7 - 12 in each group. The mRNA expression levels of TNF-α and IL-6 **(B)** were measured by qPCR to identify the effect of imipramine co-treatment (Co-Imi). The CT values were normalized as a ratio as controls being 1 and RQ value refers to the ratio of respective transcription factors as a percentage of the controls. n = 7 - 12 in each group. An immunohistochemical study was performed to determine Iba-1 immunoreactivity in the hippocampus **(C)** in 20 X (left), 40 X (right) images and cells that are immunoreactive to Iba-1 were counted in same regions of dentate gyrus. n = 10 - 15 in each group. Scale bar = 100 μm. GR levels of the hippocampus **(D)** were assessed by western blot analysis and expression of GR was quantified using Image J. n= 5 in each group. The mRNA expressions of immunologic markers **(E)** such as IL-1β, IL-6, TNF-α in the hypothalamus were measured by qPCR. The CT values were normalized as a ratio as controls being 1 and RQ value refers to the ratio of respective transcription factors as a percentage of the controls. n = 5 in each group. Iba-1 immunoreactivity was also assessed in the hypothalamus **(F)** in 4 X images. The images were taken from bregma level - 0.46 mm (left) and bregma level - 2.06 mm (right). n = 10 - 15 in each group and shown images are representative images. Scale bar = 50 μm. * p < 0.05, ** p < 0.01 compared with the controls. # p < 0. 05, ## p < 0.01 compared with the CHC- treated groups.

The hippocampus is widely known as the key brain region involved in depression [33, 38]. To identify the effect of CHC in this area, we investigated the change in inflammatory cytokines and microglia. TNF-α and IL-6 mRNA levels were increased in the hippocampus of CHC mice (Figure 3A) and were restored by co-imipramine treatment (Figure 3B). Brain-derived neurotrophic factor (BDNF), KYN pathway-related factors (IDO, KAT, KMO), microglia functional phenotype markers (CX3CR1, CD200r, p2ry12), angiotensin receptors (AT1a, 1b, 2), and serotonin receptors (5-HT1a, 2a) were not altered by CHC in the hippocampus (Figure 3A). The change in number and morphology of microglia were analyzed by immunohistochemical staining with Iba-1. Figure 3C showed that CHC increased the number of microglia (Iba-1 positive cell) in the hippocampus. Moreover, CHC mice showed more ramified microglia and the change was reversed by imipramine co-treatment. The protein level of GR in the hippocampus was also measured to identify the influence of CHC on the hippocampus. Many researchers reported that pro-inflammatory cytokine such as TNF-α can decrease glucocorticoid receptor (GR) level and result in glucocorticoid resistance [39]. Figure 3D described that CHC decreased GR protein level in the hippocampus. To identify the effect of CHC on other brain area and determine the extent of inflammatory reaction in brain, the hypothalamus of mice, which is well-known depression-related area, was analyzed with qPCR and immunohistochemistry. Figure 3E and 3F showed that CHC did not altered mRNA expressions of inflammatory cytokines and the number of microglia (Iba-1 positive cell) in the hypothalamus.

### CHC did not affect serotonin system and HPA axis in brain of mice

**Figure 4:**
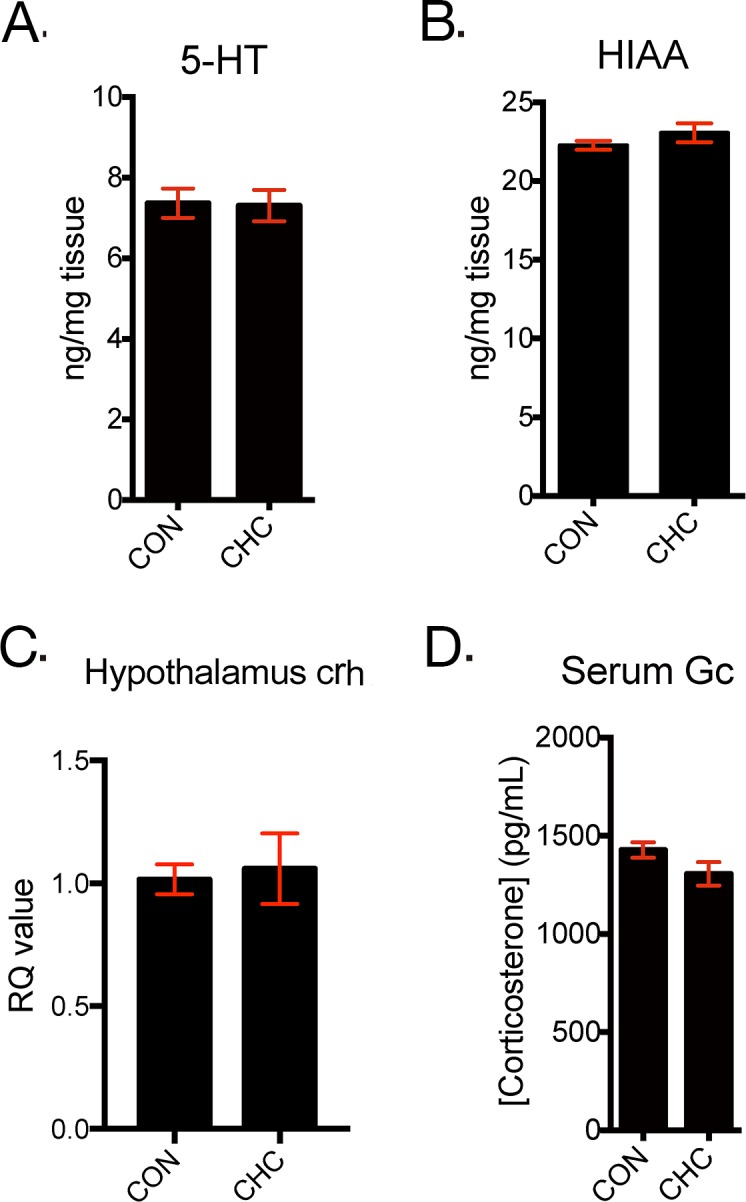
CHC did not affect monoamine system and HPA axis in brain of mice The levels of serotonin (5- hydroxytryptamine, 5-HT) **(A)** and hydroxyindoleacetic acid (HIAA) **(B)** were measured in hippocampus tissue of mice and n = 5 in each group. The mRNA expressions of CRH (corticotropin-releasing hormone) in the hypothalamus **(C)** were assessed by qPCR and n = 5 in each group. Serum glucocorticoid levels (d) were measured by EIA assay and n= 5 in each group. Data shown are mean ± SEM.

To verify the effect of CHC on other pathways related to depression, factors associated with monoamine system and HPA axis in brain were assessed. Serotonin (5-HT) and its main metabolite, 5-hydroxyindoleacetic acid (5-HIAA) were measured in the hippocampus by HPLC and showed no change in CHC mice (Figure 4A, 4B). In addition, CHC did not affect to CRH mRNA expression in the hypothalamus and serum glucocorticoid level (Figure 4C, 4D).

## DISCUSSION

In this study, CHC (40 mg/kg/day * 21 days) treated mice exhibited depression-like behaviors in only TST and FST, but not SP. Moreover, CHC did not affect anxiety and sociality. However, imipramine or captopril discontinuation rescued CHC-induced depressive-like behaviors in TST and FST. Although CHC mice exhibited less weight gain compared to the controls, it did not seem to be due to systemic illness because CHC increased glycerol release and lipid metabolism [35]. In addition, CHC did not affect to serum GOT level and motor activity. Therefore, we concluded that CHC might have distinct pathomechanism from other depression models [40, 41].

Our data suggest that depressive-like behavior induced by CHC are associated with alteration of the peripheral immune system, including elevations of pro-inflammatory cytokines such as IL-1β and IL-6, with Treg and Th2 reduction. Treg reduction induces pro-inflammatory cytokines elevation [42], which can suppress inducible Treg development, and results in a vicious cycle [43, 44]. Consistent with the alteration in the peripheral cytokine profiles, mRNA expressions of gata3 (Th2 transcription factor) and foxp3 (Treg transcription factor) were decreased in the mesenteric lymph nodes in the CHC mice. These data propose that CHC reduce Treg population and this immunologic change might elevate the peripheral pro-inflammatory cytokines.

Many investigators have suggested a correlation between peripheral cytokines and depression [15, 45]. A meta-analysis reports significantly higher concentrations of the pro-inflammatory cytokines, such as TNF-α and IL-6, in blood of depressed subjects [36]. CHC also increased serum IL-1β and IL-6 level. Assuming that this peripheral immunologic change may have an influence on brain inflammation [19, 28], we found that mRNA expressions of pro-inflammatory cytokines, such as TNF-α and IL-6, were increased in the hippocampus of CHC mice. These changes were rescued by imipramine. This result is also supported by the previous study that reported that imipramine decreased pro-inflammatory cytokines in microglial cells [46]. Captopril-treated BV-2 cells, murine microglia cell line, did not exhibit significant changes in pro-inflammatory cytokines (data not shown) and these changes in pro-inflammatory cytokines in the hippocampus may not be a direct effect of captopril on microglia. In contrast with serum level, hippocampal mRNA expression level of IL-1β was not changed in CHC mice. Based on the report that chronic stress can elevate IL-6 level in the absence of elevations of IL-1β in the cortex [47], it seems to be possible that CHC can elevate both TNF-α and IL-6 mRNA without IL-1β among pro-inflammatory cytokines in the hippocampus of CHC mice.

Considering that microglia can release pro-inflammatory cytokines, the increased number and ramifications of microglia may be associated with elevation of TNF-α and IL-6 mRNA expressions in the hippocampus [21]. In consistent with many papers suggesting association between inflammatory microglia and depression [15], inflammatory microglia might be a critical role in depressive-like behaviors in CHC mice. In addition, inflammatory microglia with TNF-α and IL-6 increase might contribute to reduction of GR expression in the hippocampus, leading to glucocorticoid resistance, which is observed in depressed patient [39]. Van Bogaert et al.[48] suggested that reduced GR levels by TNF-α may not be dependent on increased corticosterone production. However, CHC did not affect CX3CR1 and CD200r mRNA expressions in the hippocampus, which were known as anti-inflammatory functional markers of microglia related with stress vulnerability [19, 49].

Hypothalamic-Pituitary-Adrenal (HPA) axis activation is exhibited in many depression animal models and hypothalamus, a constituent of HPA axis, is one of brain areas that is often examined in depression studies. Contrary to the hippocampus, CHC did not induce inflammatory reaction in the hypothalamus. Although we could not explain exactly the region-specific inflammatory reaction in CHC mice, it might be related with fact that hippocampus is known to be one of the most vulnerable area to stressful stimuli in brain because of its high plasticity [50]. However, further study will be required to clarify the mechanism of CHC.

Our result showed that CHC-mice did not affect depression-associated factors such as BDNF, serotonin, and KYN pathway. BDNF, serotonin receptors, and IDO have been suggested as main targets of antidepressant studies [51]. However, we did not observe any significant change in mRNA expressions of BDNF, IDO, KAT, and KMO in the hippocampus of CHC mice, while they exhibited depressive-like behavior. Moreover, CHC did not induce significant change in 5-HT and HIAA concentration and expressions of serotonergic receptors in the hippocampus of mice. Furthermore, CRH mRNA expression in the hypothalamus and serum corticosteroid level was not altered in CHC mice despite reduction of hippocampal GR expression mentioned above. Thus, CHC seems to induce depressive-like behaviors through a possible alternative pathway instead of well-known mechanisms including serotonergic pathway, HPA axis activation, or KYN pathway.

Notably, we observed increased level of ANG II in CHC mice despite the chronic high-dose captopril administration. Captopril is known to decrease blood pressure by reducing synthesis of ANG II from ANG I by inhibiting ACE. This contradictory result can be explained by a possibility that chronic ACE inhibition by CHC may have activated the compensative ACE or chymase, an enzyme that performs the same function as ACE (43). However, CHC did not affect to heart chymase and lung ACE mRNA level (data not shown). Thus, it can be speculated that RAAS system including ACE and ANG II may not be as simple as we have known (37-42). Considering this complexity of RAAS system, it is difficult to elucidate underlying mechanism clearly, which is a limitation of this study.

Interestingly, imipramine did not rescue peripheral ANG II elevation and Treg reduction in CHC mice (data not shown), while imipramine restored increased TNF-α and IL-6 in the hippocampus and depressive-like behavior induced by CHC treatment. Based on the results, ACE and ANG II may be hidden depression recurrence-related factors that may not be affected by traditional antidepressants, even though we could not determine a direct link between the ANG II and depression recurrence. Although we could not determine whether chronic ANG II elevation itself can induce depressive-like behaviors, previous papers have mentioned mood-elevating effect in therapeutic dose of captopril and suggested the association between ANG II and depression [59]. Similarly, CHC induced depressive-like behavior in mice with serum ANG II elevation in our data. Endogenous ANG II increased T-cell activation, leading to production of TNF-α [60] and blocking of ACE was found to increase Treg population in the spleen [61]. Additionally, ANG II increased IL-6 release in monocytes [62] and the increased IL-6 in CHC mice might affect to Treg population in CHC mice. It has been reported that IL-6 was required for overcoming Treg suppressive function with some other cytokines [63]. Likewise, trans-signaling via the soluble IL-6 receptor abrogates the induction of Foxp3 and the generation of Treg in naïve CD4 T cells through upregulation of the TGFβ signaling inhibitor Smad7 [64]. IL-1β, as well as IL-6, is known to modulate the activation of STAT transcription factors, thereby inhibiting FoxP mRNA expression and regulating the balance of Th17 and Treg cells [43]. These findings suggest that ANG II, IL-6, and IL-1β might be involved independently or together in reduction of Treg population. However, considering the existence of ANG II receptor in brain [59] and ability of ANG II to activate microglia and induce inflammation [65–67], it should not be excluded that increased serum ANG II by CHC may have a direct effect on alteration of mice behavior, although ANG II receptors was not changed in the hippocampus. Additionally, we cannot exclude the possibility of captopril crossing the blood-brain barrier to have a direct effect on the brain of mice through ACE or renin-angiotensin system within the brain, although captopril did not affect to mRNA level of inflammatory cytokines in BV2 cell line (data not shown). Thus, our results might be translated to explain the comorbidity with cardiovascular disease and depression [68].

In summary, we found that CHC induced a decrease in Treg population and alterations in peripheral pro-inflammatory cytokines. These changes in the peripheral immune condition might have affected the CNS, especially the hippocampus, including microglial status and expressions of pro-inflammatory cytokines, and they altogether may have led to depressive-like behavior in mice. However, CHC did not affect serotonin level in the hippocampus, KYN pathway, and HPA axis activation, which are well-known depression-related pathways. Thus, CHC may propose a novel alternative mechanism in depression pathogenesis.

## MATERIALS AND METHODS

### Experimental animals

Male C57BL/6 mice at age of 7 weeks (Orient Bio Inc. Seoul, Korea) weighing 20–25 g were used for all the experiments. Animals were housed five per cage in a room maintained at 22 ± 0.5°C with an alternating 12-h light–dark cycle. Food and water were available *ad libitum*. Animals were allowed to acclimate to the laboratory for a week before the beginning of the experiments. To reduce variation, all experiments were performed during the light phase of the cycle. All experimental procedures were approved by the Animal Care and Use Committee of the CHA University (IACUC130018 and IACUC130025).

### Captopril and drug treatment

Mice were randomly assigned to controls or experimental groups. For restraint stress, the mice were forced into 50 mL Corning tubes with a nose-hole for ventilation, 2 h per day (11:00 AM–1:00 PM). The mice were exposed to restraint stress (2 h/day) for 21 consecutive days and this procedure is referred to as chronic restraint stress (CRS). CRS group was added as a positive control. To determine dose-dependent effect of captopril administration, captopril (Sigma-Aldrich, St. Louis, Missouri) was administered by dissolving in their drinking water at concentration of 25 or 40 mg/kg/day. To examine time-course effect of captopril, 40 mg/kg of captopril was administered in their drinking water for 7 days, 14 days and 21 days. The daily drinking volume of control mouse was 4.216 ± 0.1375 ml and that of CHC mice was 3.92 ± 0.1013 ml. There was no significant difference in daily drinking volume between control and CHC-mice.

***Experiment 1:*** Following the examination of both dose-dependent effect and time-course effect of captopril, chronic high-dose captopril (CHC) was defined as 40 mg/kg/day of captopril treatment for 21 days or more, and captopril treatment was continued during period for behavior assessment (Supplementary Figure 1A).

***Experiment 2:*** In order to evaluate the effect of captopril washout, captopril in drinking water was removed and regular drinking water was given for 7 days after the termination of 21 day of captopril treatment (Supplementary Figure 1B).

***Experiment 3:*** To assess antidepressant effect of imipramine co-treatment (Co-Imi), imipramine (Sigma-Aldrich, St. Louis, Missouri; 20 mg/kg) was dissolved in physiologic normal saline and treated intraperitoneally once a day during captopril administration (Supplementary Figure 1C).

***Experiment 4:*** In order to assess antidepressant effect of imipramine after induction of depression-like behavior by CHC, imipramine was treated for 14 days with additional captopril after 21 days of captopril treatment (post-Imi) (Supplementary Figure 1D).

### Behavioral testing

Behavior testing was performed referring to our previous studies [19, 33]. Mice were allowed to acclimate to a testing room for at least 30 min before performing the experiments. All experiments were conducted during the light cycle between 9:00 AM and 4:00 PM in a series, one experiment per day. Light-dark exploration (LD), elevated plus maze (EPM) and SI were done using EthoVision XT9 video tracking system (EthoVision® Version 9, Noldus, Netherlands). Tail suspension test (TST) and forced swimming test (FST) were conducted by two observers to minimize error.

*Rotarod test:* Locomotor activity of mice was assessed using a rotarod system (Rota Rod-R V2.0, B.S Technolab INC). Before measuring, mice were adapted to the rod with a constant speed of 4 rpm for 3 minutes. Mice were conditioned on the rod (diameter: 3.5 cm) with an increasing speed of 4 to 40 rpm (accelerated by 1 rpm per 5 s). Motor ability was measured as the time until the mouse falls off the rod, up to 300 s. The average latency time of 3 trials was calculated for statistical analyses. The interval period between trials was 20 min.

*Sucrose preference test (SP):* Preference for sucrose solution over drinking water was measured to assess anhedonia, which is usually decreased in depressed mice as same as previous paper [19]. To assess SP, mice were provided with two bottles filled with 1 % sucrose diluted in drinking water or drinking water alone. Animals were acclimatized to two bottle conditions for two consecutive days and were tested for their choice for two additional days. The position of the bottles was interchanged during 4 days of testing. On each test day, the fluid levels were noted. SP was calculated as percentage of sucrose/total fluid consumed.

*Light–dark exploration (LD):* Anxiety-like behavior was measured using EPM and LD by assessing their tendency to avoid bright light and open spaces. The apparatus used in this assessment was a box (30 × 30 × 30 cm) consisting of one brightly lit open chamber connected to a darkened enclosed chamber. The chambers were connected by a small square hole (7×7 cm). Mice were placed in the corner of the lit chamber, facing away from the dark chamber, and the number of transitions between the chambers and time spent in the dark chamber were manually measured for 10 min.

*Elevated plus maze (EPM):* EPM apparatus and procedure were modified as our previous paper [19]. The apparatus consisted of four open roof arms (30 × 5 cm) made of white matte plexiglass. The two opposite arms were enclosed with 20-cm-high walls (closed arms), and the remaining two opposite arms had no walls (open arms). The four arms were placed at 90° to each other around a 5 × 5-cm square in the center. The apparatus was elevated 30 cm above the floor. The mouse was initially placed in the center of the apparatus, facing one of the open arms away from an experimenter, and allowed to explore the apparatus freely for 5 min. The number of entries to open arms and closed arms was recorded, and the times spent in each arm were recorded using EthoVision XT9 video tracking system.

*Tail suspension test (TST):* The apparatus consisted of a cupboard with a hook attached to the top. The mice were suspended by securing the tail to the hook by wrapping adhesive tape around the tail. The tail was suspended carefully not to fold the tail, and the tip of the tail was wrapped 2 cm away from the top of the hook. The data of the mice that climbed their tails were removed from the test. The time spent immobile during a 7-min testing period was measured. The observers were blinded to the groups. The time spent immobile was measured and compared by two observers to minimize the bias.

*Forced swimming test (FST):* In FST, we assessed the ability of mice to cope with an inescapable stressful situation, which reflects depressive-like behavior. Mice were individually placed in a 2 L Pyrex beaker (13 cm diameter, 24 cm height), filled with 23°C water with a depth of 17 cm. All mice were forced to swim for 6 min, and the duration of immobility was measured during the final 5 min of the test. The immobility was defined as the time that the mouse spent floating without struggling and making only the movements necessary to keep its head above the water level. The observers were blinded to the groups. The time spent immobile was measured and compared by two observers to minimize the bias.

*Social interaction (SI):* Social interaction was performed as previously described [34]. In trial 1 (Empty), mice were placed into the arena with empty wire mesh cage at one end. Their movement was monitored for 2.5 min in the absence of a mouse. In trial 2 (Social), an unfamiliar mouse was placed in the wire mesh cage and the experimental mouse was placed in the arena, and activity was recorded for 2.5 min. Activity in the social avoidance behavior test was video recorded and analyzed using EthoVision XT9 video tracking system.

### Flow cytometry analysis

Spleen was digested with collagenase IV (Sigma-Aldrich, St.Louis, MO) and DNase I (Sigma-Aldrich) in RPMI medium (Gibco, Carlsbad, CA) for 30 min at 37°C, and filtered through 40 μm Gauze (BD Biosciences, San Jose, CA) to generate single cell suspension. Cells were fixed, permeabilized, and stained mouse regulatory T cell staining kit (eBioscience, San Diego, CA, 88-8118-40). The regulatory T cell were measured and analyzed according to the manufacturer's instruction. Briefly, 5 ×10^6^/mL of cells were immunolabeled using antibodies against surface proteins CD4, CD25, and intracellular protein FoxP3. Briefly, cells were incubated in CD4-FITC and CD25- PE or isotype-matched control antibodies for 20 min at 20-23.5°C in the dark; the cells were then washed with 1 mL staining buffer and fixed with the fixation buffer that was supplied in mouse regulatory T cell staining kit for 10 min at 20-23.5°C in the dark. The cells were permeabilized using permeabilization buffer for 1 h at 20–23.5°C and then washed with 1 mL of staining buffer. After washing, the cells were incubated in APC-conjugated anti-mouse FoxP3 or the isotype control for at least 30 min at 20–23.5°C in the dark. At the end of the incubation period, cells were washed and resuspended in staining buffer. Data acquisition was performed with BD FACS Calibur and data were analyzed with FlowJo software (Treestar Inc., Ashland, OR).

### Quantitative reverse transcriptase polymerase chain reaction (qRT-PCR)

The hippocampus, hypothalamus and the mesenteric lymph nodes were dissected after captopril administration for 21 days followed by series of behavioral test. For RNA extraction, the frozen tissue was homogenized in 1 mL of QIAzol reagent per 100 mg of tissue (Qiagen, Valencia, CA). Chloroform was added to separate the phase that contains RNA, and isopropyl alcohol was added to precipitate RNA. The precipitated RNA pellet was re-dissolved in DEPC- treated water (Bioneer, Seongnam, Korea) after air-drying the pellet. Quantification of RNA concentration was determined by the absorption at 260 nm. One microgram of messenger RNA (mRNA) was reverse-transcribed into cDNA in 20 μL of reaction mix using RevertAid First Strand cDNA Synthesis kit (Thermo scientific). Quantitative PCR was performed using Power SYBR® Green PCR Master Mix (Life technologies, Warrington, UK). Primer sequences are listed in Table 1. The cyclic conditions consisted of an initial enzyme activation at 95°C for 5 min followed by 40 cycles of denaturation at 95°C for 20 s, annealing, and extension including detection of SYBR Green bound to PCR product at 56°C for 40 s. Glyceraldehyde 3-phosphate dehydrogenase (GAPDH) was used as an internal control for normalization. The relative quantities of PCR fragments were calculated using the comparative CT method.

**Table 1:** Primer information

Primer	Forward (5′ → 3′)	Reverse (5′ → 3′)	PCR product(bp)
Tbx21	CAACAACCCCTTTGCCAAAG	TCCCCCAAGCAGTTGACAGT	108 bp
Gata3	AGAACCGGCCCCTTATCAA	AGTTCGCGCAGGATGTCC	71
Foxp3	GAACCCAATGCCCAACCCTAG	TTCTTGGTTTTGAGGTCAAGGG	1311
BDNF	TGCAGGGGCATAGACAAAAGG	CTTATGAATCGCCAGCCAATTCTC	109
IDO	TGTGAATGGTCTGGTCTC	CTGTGCCCTGATAGAAGT	238
KAT	GTTCTCCACACACAAGTCTC	GGATCCATCCTGTCAGTCA	524
KMO	GGTCGCCTTCACCAGAATAA	ATCCAGGCAGGTCTTCTCAA	176
TNF-α	GAGTCCGGGCAGGTCTACTTT	CAGGTCACTGTCCCAGCATCT	234
IL-1β	GGCTGGACTGTTTCTAATGC	ATGGTTTCTTGTGACCCTGA	133
IL-6	CCACTTCACAAGTCGGAGGCTTA	GCAAGTGCATCATCGTTGTTCATAC	111/172
5-HT1A	CTGTTTATCGCCCTGGATG	ATGAGCCAAGTGAGCGAGAT	157
5-HT2A	CCGCTTCAACTCCAGAACCAAAGC	CTTCGAATCATCCTGTACCCGAA	108
AT1aR	GGACACTGCCATGCCCATAAC	TGAGTGCGACTTGGCCTTTG	144
AT1bR	CTGCTATGCCCATCACCATCTG	GATAACCCTGCATGCGACCTG	147
AT2R	GCACCAATGAGTCCGC	AGGGAGGGTAGCCAAA	214
Cx3cr1	TGGCCCAGCAAGCATAG	CATGTCTGCTACCCTCACAAA	93
Cd200r	AAATGCAAATTGCCAAAATTAGA	GTATAGCTAGCATAAGGCTGCATTT	73
P2ry12	GGGCGTACCCTACAGAAACA	TGTTGACACCAGGCACATCC	204
CRH	GTTAGCTCAGCAAGCTCACAG	GCCAAGCGCAACATTTCATTT	69

### Immunohistochemistry

For perfusion, mice were sacrificed after CHC treatment. All mice were first deeply anesthetized with pentobarbital (100 mg/ kg, i.p.), and perfused through the heart with physiological saline followed with ice-cold phosphate-buffered 4% paraformaldehyde (pH 7.4). Whole brain was dissected and post-fixed in the same fixative for 4 hr at 4°C. Then the brain blocks were cryoprotected in 30% sucrose for 24 hr at 4°C. Sections were cut with an electronic cryotome at a thickness of 25 mm. Immunohistochemical staining was performed with the Elite ABC Kit (Vector Laboratories). Sections were first rinsed with 0.1 M bovine serum albumin three times for 10 min each, then pre-incubated in 0.1 M PBS containing 1% bovine serum albumin and 0.2% Triton X-100 for 30 min. After rinsing twice with 0.1 M PBS containing 0.5% BSA for 10 - 15 min each, sections were incubated with anti-Iba-1 antibody (1:300; Wako, Cat. # 019-19741) diluted with 0.1 M PBS containing 0.5% BSA and 0.05% sodium azide at 4°C. After overnight incubation, sections were rinsed and incubated with biotinylated anti-rabbit IgG secondary antibody (Vector) 1:200 diluted with 0.1 M PBS containing 0.5% BSA for 1 hr at room temperature. After rinsing, the sections were incubated with ABC reagent 1:200 diluted with PBS for 1 hr at room temperature and then rinsed with PBS followed with 0.1 M phosphate buffer. Finally, sections were incubated in SIGMA FAST DAB kit (Sigma) until the desired stain intensity developed. Sections were rinsed with 0.05 mol/L phosphate buffer, and then the sections were dehydrated through graded ethanols, cleared in histoclear (Fisher), and cover slipped using Permount (Fisher). Histological analysis was performed using the following procedure, which is modified from the previous studies [33, 69]. The number of Iba-1-positive neurons in the hippocampus was counted in three sections for each mouse. Starting from the first section (interaural 2.10 mm, bregma −1.70 mm), counts were taken from three coronal sections at 0.135 mm increments. The number of cells that are immunoreactive to Iba-1 was counted by two blinded observers using a microscope in the same brain area (Nikon). For images of hypothalamus, sections were taken from the level of interaural 3.34 mm, bregma – 0.46 mm to the level of interaural 1.50 mm, bregma – 2.30 mm at 0.135 mm increments.

### Total protein extraction and western blot analysis

Hippocampal protein was extracted and expression levels were assessed using Western blotting. After dissecting the hippocampus, the tissue was washed two times with cold Tris-buffered saline (TBS; 20-mM Trizma base and 137 mM NaCl, pH 7.5). Immediately after washing, tissue was lysed with SDS lysis buffer (62.5 mM Trizma base, 2 % w/v SDS, 10 % glycerol) containing 0.1 mM Na3VO4, 3 mg/ml aprotinin, and 20 mM NaF. After brief sonication to shear DNA and reduce viscosity, protein concentration was determined with a detergent-compatible protein assay reagent (Bio-Rad Laboratories) using bovine serum albumin as the standard. After adding dithiothreitol (5 mM) and bromophenol blue (0.1 % w/v), the proteins were boiled, separated by electrophoresis in 10 % polyacrylamide gels (Invitrogen), and transferred onto a polyvinylidene difluoride (PVDF) membrane (Bio-Rad Laboratories). Membranes were blocked on a shaker for 1 h at room temperature. Blocking buffer consisted of TBST (Tris-buffered saline/0.1 % Tween-20) and 5 % skim milk. Primary antibodies were dissolved in the blocking buffer and the membranes were immunoblotted with antibodies against glucocorticoid receptor (GR; 1:1000, Santa cruz, Sc-1004) and beta-actin (1:1000, Cell Signaling, 4970) for overnight at 4°C. The membranes were incubated in the anti-rabbit (1:2000, Cell Signaling, 7074) dissolved in the blocking buffer at a room temperature for 80 min. The membranes were visualized with ECL-plus solution (Amersham Pharmacia Biotech). Then, the membranes were then exposed to chemiluminescence (LAS- 4000, Fujifilm) for detection of light emission. Western blot results were quantified using ImageJ 1.51 software (National Institutes of Health, Bethesda, MD) after densitometric scanning of the films.

### Quantification of serotonin (5-hydroxytryptamine, 5-HT) and its metabolite, 5- hydroxyindoleacetic acid (5-HIAA) by high pressure liquid chromatography and electrochemical detection (HPLC-ECD)

Mice were immediately sacrificed after captopril administration for 21 days followed by series of behavioral test. Brain tissue blocks were rapidly dissected in ice and homogenized with ice-cold 0.4M perchloric acid, then incubated on ice for 1 h. After centrifugation at 21,130 g for 30 min at 4°C, the supernatant was filtered using an appropriate column (#Sc1000-1Kt, SigmaPrep spin column). Filtered supernatants were directly injected onto the Nova-Pak C18 reversed-phase column. The mobile phase consisted of 0.1M sodium phosphate monobasic, 0.1mM EDTA, 1mM sodium octyl sulfate, 0.003% trimethylamine, and 10% methanol at pH 3.7. The external standard solutions for each analyte were 5-hydroxytryptamine (5-HT; sc-298707, Santa Cruz) and 5-hydroxyindole-3-acetic acid (5-HIAA; #H8876, Sigma-Aldrich) solutions in HPLC grade water with 0.4M perchloric acid. The flow rate was kept constant at 1 ml/min. Chromatographic peak analysis was accompanied by identification of unknown peaks in a sample matched according to retention times.

### Determination of serum chemistries

Mice were sacrificed after captopril administration for 21 days followed by series of behavioral test and whole blood was collected by cardiac puncture. The serum was isolated and stored at −80°C until assayed.

Serum oxaloacetic transaminase (GOT) level was measured by Reitman-Frankel method with GOT kit according to manufacturer's instruction (Asan pharmaceutical, Seoul, Korea).

Serum cytokine (TNF-α, IL-1β, IL-6, IL-17, IL-4, IL-10 and IFN-γ) levels were measured using Bio-Rad Bio-Plex® assay (Bio-Rad, Hercules, CA). The cytokine levels were measured and analyzed according to the manufacturer's instruction.

Serum angiotensin II (ANG II) level was measured by angiotensin II EIA kit according to manufacturer's instructions (Pheonix Pharmaceuticals, Burlingame, CA, USA).

Serum corticosterone level was determined by corticosterone EIA kit according to manufacturer's instructions (Cayman Chemical, Ann Arbor, MI).

### Statistical analysis

Data were presented as the mean ± standard mean error (SEM). The statistical significance of differences between groups was assessed with Student's t test and one-way analysis of variance (ANOVA) using GraphPad Prism version 7 for Mac (GraphPad, La Jolla, CA). Tukey's post hoc test was performed when p values were <0.05. p < 0.05 was considered as statistically significant.

## SUPPLEMENTARY MATERIALS FIGURES



## Abbreviations

CHC: chronic high dose captopril (40 mg/kg/day *21 days or more)
TST: tail suspension test
FST: forced swimming test
LD: light-dark exploration
EPM: elevated plus maze
SI: social interaction test
Treg: regulatory T cell
Th2: type 2 T helper cell
IL: interleukin
TNF: tumor necrosis factor
ANG II: angiotensin II
ACE: angiotensin-converting enzyme
CNS: central nervous system
IFN: interferon
KYN: kynurenine
BDNF: brain-derived neurotrophic factor
IDO: indoleamine 2,3-dioxygenase
KAT: kynurenine aminotransferase
KMO: kynurenine 3-monooxygenase
NMDA receptor: N-methyl-D-aspartate receptor
LPS: lipopolysaccharide
CRS: chronic restraint stress
Co-Imi: co-treatment of imipramine
Post-Imi: post-treatment of imipramine
GOT: glutamic oxaloacetic transaminase
GR: glucocorticoid receptor
qPCR: real-time polymerase chain reaction
HPA axis: hypothalamic-pituitary-adrenal axis
5- HT: 5-hydroxytryptamine
5-HIAA: 5-hydroxyindoleacetic acid
CRH: corticotropin-releasing hormone
