# Supplementary material for: Chronic high dose of captopril induces depressive-like behaviors in mice: possible mechanism of regulatory T cell in depression
Source: Oncotarget. 2017 Aug 3;8(42):72528–43. doi: 10.18632/oncotarget.19879 (PMC5641150; doi:10.18632/oncotarget.19879)
Supplement: Supplementary file 1 [file oncotarget-08-72528-s001.pdf]

Chronic high dose of captopril induces depressive-like behaviors in mice: possible mechanism of regulatory T cell in depression

SUPPLEMENTARY MATERIALS

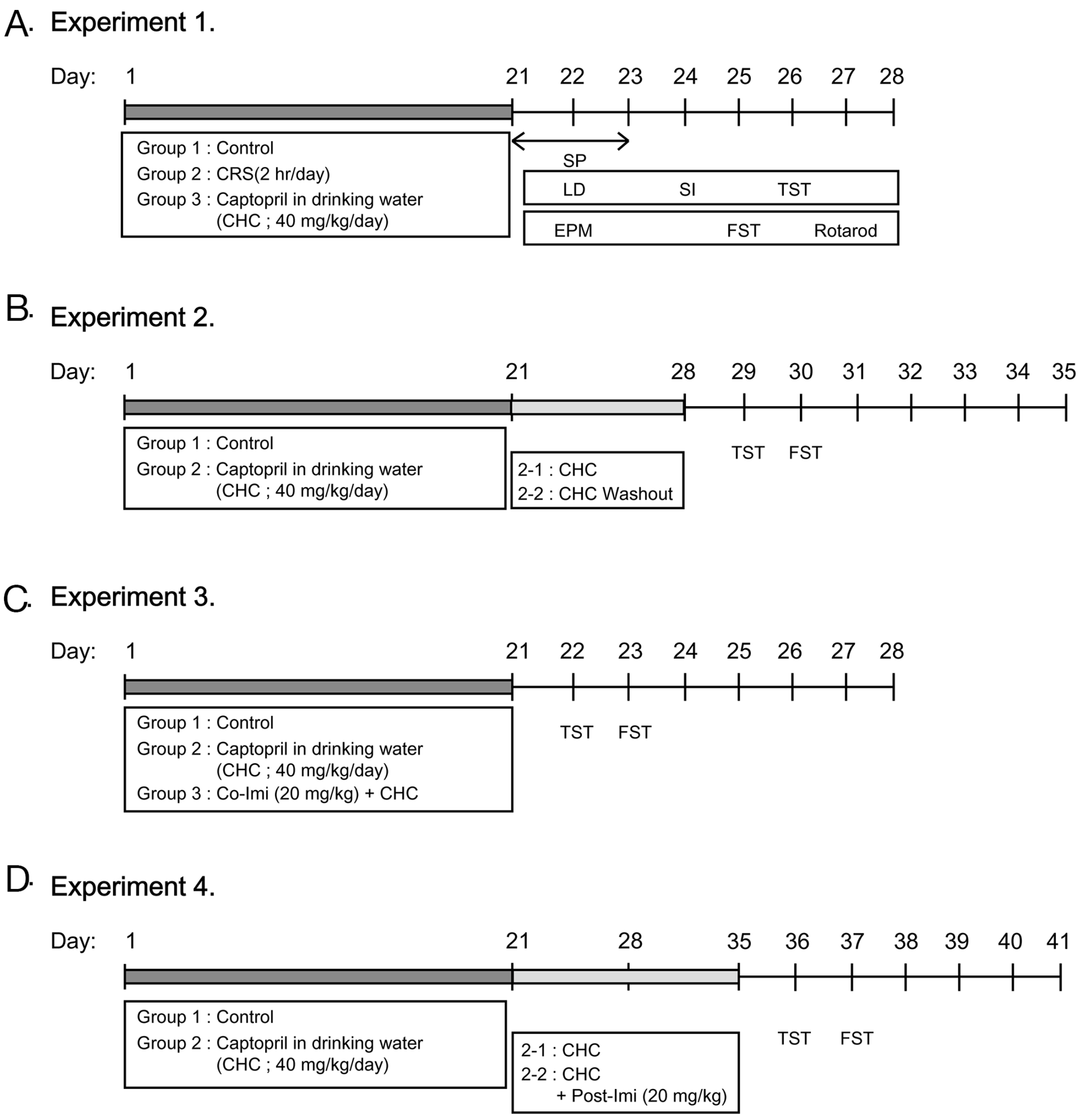

Supplementary Figure 1: Timeline of experimental schedule. Chronic high-dose captopril (CHC) is defined as captopril administration (40 mg/kg) for 21 days or more and captopril administration was continued during behavioral tests. To identify the effect of captopril on behavior, mice were randomly assigned to the control, chronic restraint stress group (CRS) and CHC group **(A)**. Mice in each group were divided into 2 groups and we performed different set of behavior tests in each subgroup. The behavior test including TST, FST, SP, LD, EPM, SI and rotarod were performed to assess behavior of mice associated with depression, anxiety and sociality. To assess captopril washout effect, regular drinking water without captopril was provided to mice after 21 days of captopril administration for following 7 days and TST and FST were performed within 1 weeks since captopril washout **(B)**. To identify the effect of antidepressant on CHC-induced depressive-like behavior, imipramine (20 mg/kg) was administrated intraperitoneally once a day with captopril administration (Co-Imi) and depressive-like behavior of mice were assessed by TST and FST **(C)**. In addition, imipramine at same dose was treated after captopril administration for 21 days (post-Imi) and depressive-like behavior of mice were assessed by same tools with Co-Imi **(D)**.

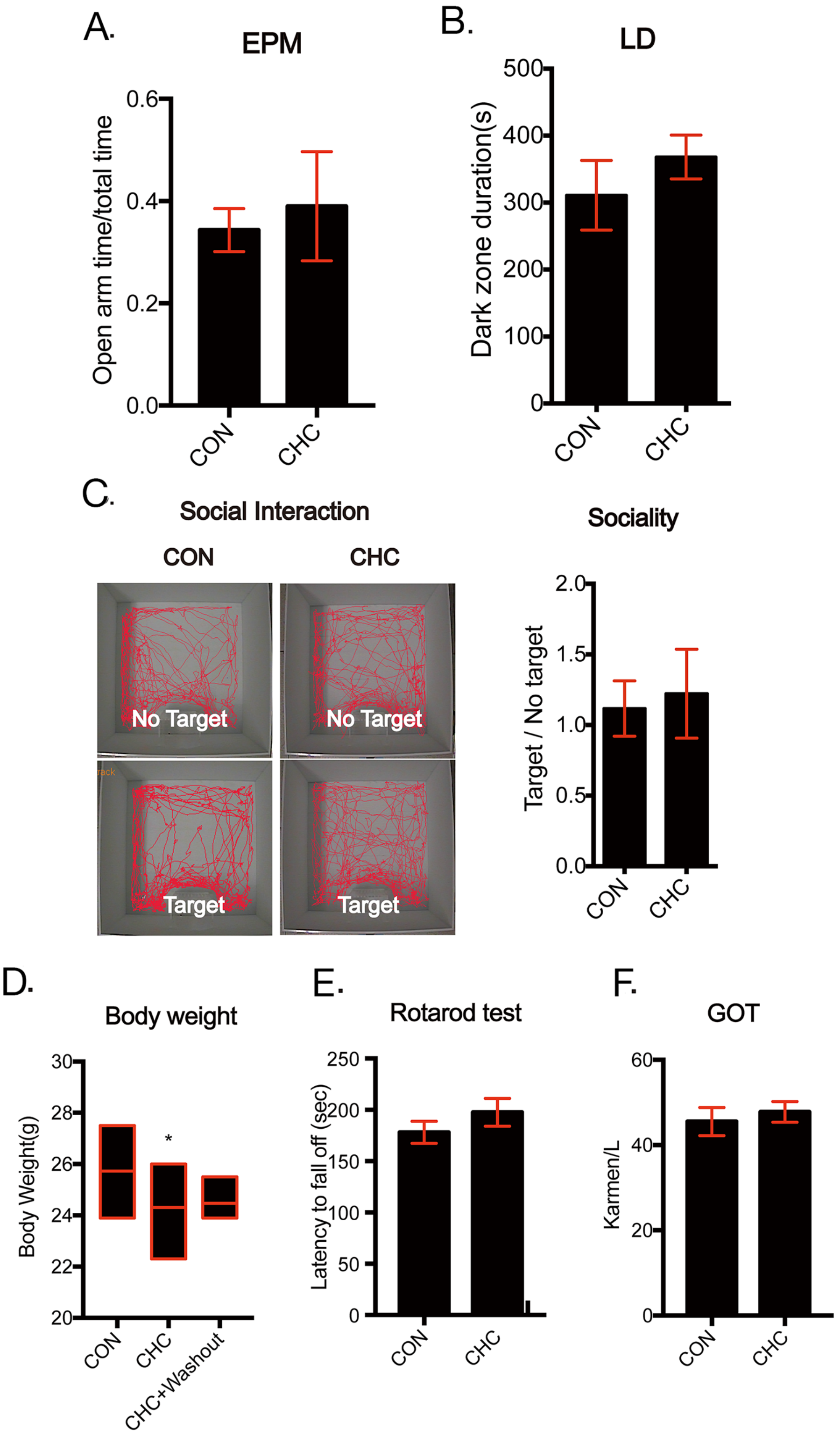

Supplementary Figure 2: CHC did not affect anxiety, sociality and general condition in mice. Anxiety-like behaviors in CHC mice were assessed by elevated plus maze (EPM), light-dark exploration (LD). EPM: the time spent in the open arms was measured and was expressed as a ratio **(A)**. LD: the time spent in the dark zone during the 10 min was measured **(B)**. Social interaction tests were performed in CHC mice and controls using EthoVision XT9 and data were also expressed to the ratio of target to no target **(C)**. n = 7 - 10 in each group and the data shown are mean ± SEM. Body weight **(D)**, serum GOT level **(E)** were measured and rotarod **(F)** test were performed to evaluate general condition of mice and general toxic effect of captopril and n = 10 - 15 in each group. Data shown are mean ± SEM. $^*p < 0.05$ compared with the controls.
